# Indents

**Published:** 1912-08-15

**Authors:** 


### INDENTS.

☞Brief Articles of Technical or Scientific Value will receive notice and comment. Contributions invited.

*Importance of Anesthesia in Treatment of Inflammation.* Spiess and Feldt have been conducting researches to confirm still further Spiess' assumption that the main factor in inflammation is the work of the sensory nerves, and that consequently means to exclude the action of the sensory nerves is the logical first step in treatment. This can be done by anesthetizing them or by making it impossible for them to exert any reflex action. Inflammation, they affirm, is a reaction on the part of the vessels—a reflex process. Spiess has published a number of works on the subject, especially on application of the principle to treatment of whooping-cough (1901), coryza, etc. The redness, heat, swelling and pain of an inflammatory process are all the work of the sensory nerves, and are thus of secondary reflex nature, the vasodilatation and changes in the vessel walls being the result of the primary irritation of the afferent nerve terminals. When these nerve terminals are rendered insensible to the primary irritation, no reflex action follows and thus there is no inflammation. The inflammation once installed, the sensory nerves keep it up by a kind of vicious circles If the afferent sensory nerves at any time are anesthetized the vicious circle is broken up and the inflammation heals. The nerves need to be deadened only just enough to arrest the reflex action, and the anesthesia should affect only the sensory nerves. Animal experimentation reported by Bruce last year has confirmed Spiess' theory, as he found it impossible to induce inflammation in an anesthetized area even with agents which under other conditions induce a lively inflammation. Even mustard oil did not induce inflammation in rabbits' eyes as long as the innervation was under influence of an anesthetic or the sensory terminals were excluded from the ganglion by the nerve being severed and a stretch degenerated, thus restricting the reflex arc to the periphery. The importance of anesthetics for combating inflammation is scarcely realized, as yet, Spiess and Feldt declare, and new horizons are opening for treatment. Haug has been applying the principle in treatment of middle-ear disease since 1906 and Levinstein in the throat since 1910.—*Journal A. M. A.*, June 29, 1912.

*Arsenical Necrosis?* I would like to relate a case that came under my observation; I think possibly it may have been arsenic poisoning. I think it is well to relate the case, too, for the reason that it will lead a great many of the men who are taking up some of those base metals, alloy, etc., in filling teeth, to think about it. This was a case of Dr. Barber's, who is associated in the same suite with Dr. Reid and myself. He restored teeth in which he was going to put on a bridge. He had three cases of this kind. He restored the teeth by means of a metal known as bore metal, which is largly composed of aluminum. He built the teeth up and then prepared his crowns for his bridges, he made the bridges and set them. In the meantime, while he was constructing the bridge, the bore restoration metal was cemented in and remained in the mouth for a considerable length of time without any apparent deleterious effect. Almost immediately after placing in the mouth the bridge there was a distinct devitalization or deadening of the soft tissues entirely down to the alveolar process and back of it as large as the end of my finger, almost half an inch in length. A white, cheesy, hard mass dropped out until you could see the bone bare, absolutely denuded of periosteum and all the soft tissues. There were three of these cases that occured in his practice. It seemed to have been due to one particular kind of cement he was using. Whether it was due to the electrical or chemical effect, or whether it was due to the arsenic in the cement that it was set with, or what the condition was that caused this, was a question in the minds of all of us who saw the case.—Dr. Roach, in *Review*.

*Casting Against Pick-Ups.* I wish to tell you one more principle about gold, and casting it to other pieces of gold which are in the flask.

Without any experience except that gained by blow-pipe work, the first thought which seems to come to dentists when they wish to cast against gold is to bring both the golds to as near the same temperature as possible. This process, gentlemen, is absolutely defeating the object sought. Why?

In bringing the flask and its contents up to a bright red heat you bring the enclosed metal to a temperature at which it oxidizes, and, having no means of deoxidizing this surface, you do not get as perfect a union when the melted gold is thrown in as you will if you heat the case up to a temperature just sufficient to burn out the wax. And this temperature does not absolutely burn all the carbon off the enclosed gold, and as carbon is one of the very best deoxidizers, you now have your gold in the very best condition to be welded to other gold. Now, the thing to do, in order to cast to this gold, is to bring your melted metal up to an excessive temperature, and you will cast in a scientific manner.—W. H. Taggart, in *Review.*

*Charcoal in Treatment of Internal Disease.* Adler refers among others to Wiechowski's experiments (1909) which showed that animals given otherwise fatal doses of poisons survivèd when they were treatd with animal charcoal which seemed to absorb the poison and thus render it harmless. Adler has been applying the same measure systematically in various internal diseases and the favorable results justify, he declares, further work in this line. He advises having the charcoal in the proper doses constantly on hand so as to give it at once when need arises. In poisoning cases a suspension of the charcoal in water might be poured into the stomach and pumped out again at once as absorption occurs almost instantaneously. Then after rinsing out the stomach with water to which the ordinary antidote may be added,he advises giving a suspension of the charcoal in a solution of magnesium sulphate, if there is no contra-indication to a purge. This charcoal suspension can be given several times during the same day or kept up for several days. An enema of water in which charcoal is suspended will clear out the bowels below, so that the upper part will be more readily evacuated. He thinks there is every reason to believe that this simple and harmless method will materially reduce the mortality from poisonings. He has applied it in six cases with the best results; the poison had been phosphorus, mercuric chlorid, Paris green, tincture of opium or phloroglucin, all taken with suicidal intent. Adler suggests that the charcoal might prove useful in cholera, for typhoid-bacilli carriers, in gastro-enteritis, typhoid fever, etc. In normal conditions the charcoal seems to be inert, that is, it does not tend to induce constipation nor act as a laxative, but when the gastro-intestinal tract is being irritated and responds with diarrhea, the charcoal, by absorbing the toxins and thus keeping them away from the tissues, puts an end to the irritation and the diarrhea is arrested, The charcoal may thus serve to differentiate the cases in which the trouble is in the contents of the bowels rather than in the walls or due to nervous influences.—*Journal A. A.M.*, June 29, 1912.

*Inhalone.* Liquid inhalants containing phenol and various other medicaments soluble in oils are extensively prescribed at the present time. However, there is a large demand for a similar product of ointment consistence, which Inhalone successfully fills.

Inhalone is composed of phenol, eucalyptol, and menthol, in a petrolatum base, and is supplied in a collapsible tube with elongated tip to facilitate its introduction into the nostril. It is not in the least objectionable to the sense of smell; quite the contrary. The antiseptic action of the carbolic acid in acute and chronic catarrh is especially valuable when combined with the well-known therapeutic effects of eucalyptol and menthol. This combination stimulates the secretion of mucus, allays irritation and inflammation, and improves the nasal respiration, frequently within a few hours.

